# Integrated Metabolomics and Transcriptomics Analyses Reveal the Regulatory Mechanisms of Anthocyanin and Carotenoid Accumulation in the Peel of *Coffea arabica*

**DOI:** 10.3390/ijms251910754

**Published:** 2024-10-06

**Authors:** Zuquan Wang, Chun Xie, Yihong Wu, Haobo Liu, Xuesong Zhang, Huabo Du, Xuejun Li, Chuanli Zhang

**Affiliations:** College of Tropical Crops, Yunnan Agricultural University, Pu’er 665000, China; 2023240805@stu.ynau.edu.cn (Z.W.); 2008077@ynau.edu.cn (C.X.); 2024240849@stu.ynau.edu.cn (Y.W.); 2024240846@stu.ynau.edu.cn (H.L.); 2020240160@stu.ynau.edu.cn (X.Z.); 2000047@ynau.edu.cn (H.D.)

**Keywords:** *Coffea arabica*, fruit peel coloration, ripening process, anthocyanins, carotenoids, metabolomics, transcriptomics

## Abstract

The color of coffee fruits is influenced by several factors, including cultivar, ripening stage, and metabolite composition. However, the metabolic accumulation of pigments and the molecular mechanisms underlying peel coloration during the ripening process of *Coffea arabica* L. remain relatively understudied. In this study, UPLC-MS/MS-based metabolomics and RNA sequencing (RNA-seq)-based transcriptomics were integrated to investigate the accumulation of anthocyanins and carotenoids in the peel of *Coffea arabica* at different ripening stages: green peel (GP), green-yellow peel (GYRP), red peel (RP), and red-purple peel (RPP). This integration aimed at elucidating the molecular mechanisms associated with these changes. A total of ten anthocyanins, six carotenoids, and thirty-five xanthophylls were identified throughout the ripening process. The results demonstrated a gradual decrease in the total carotenoid content in the peel with fruit maturation, while anthocyanin content increased significantly. Notably, the accumulation of specific anthocyanins was closely associated with the transition of peel colors from green to red. Integrated metabolomics and transcriptomics analyses identified the GYRP stage as critical for this color transition. A weighted gene co-expression network analysis (WGCNA) revealed that enzyme-coding genes such as 3AT, BZ1, and lcyE, along with transcription factors including MYB, NAC, and bHLH, which interact with PHD and SET TR, may regulate the biosynthesis of anthocyanins and carotenoids, thereby influencing peel pigmentation. These findings provide valuable insights into the molecular mechanisms underlying the accumulation of anthocyanins and carotenoids in *Coffea arabica* peel during fruit maturation.

## 1. Introduction

Coffee plants belong to the genus *Coffea* of the *Rubiaceae* family, encompassing over 130 recognized species, and are widely cultivated as economically significant crops worldwide [1]. According to the International Coffee Organization’s report, global coffee production increased by 0.1% in the 2022/23 season, totaling 168.2 million bags, and is projected to increase by 5.8% to 178 million bags in the 2023/24 season [2]. A key issue in research on coffee is that coffee contains hundreds of other biologically active phytochemicals, including polyphenols such as chlorogenic acid and lignans, the alkaloid trigonelline, melanoidins formed during roasting, and modest amounts of magnesium, potassium, and vitamin B3 (niacin) [3]. The phytochemical profile of coffee is affected by genetic traits, cultivar, environment, growing condition, level of maturation, processing conditions, and pre- and post-harvest handling practices [4]. In the coffee-processing workflow, tissues such as coffee peels and pulps are often discarded in large quantities owing to their limited utilization. However, these byproducts are rich in bioactive compounds like polyphenols, carotenoids, and anthocyanins, which exhibit significant nutritional value and medicinal potential [5,6]. Particularly, *Coffea arabica* L., one of the premium coffee varieties in the international market [7], warrants further investigation of the mechanisms underlying natural pigment changes in its peels. Although previous studies have primarily focused on coffee cultivation techniques [8], roasting processes [9], chemical composition analyses [10], and flavor quality [11], research on the dynamic regulatory mechanisms of husk pigment accumulation, particularly the synthesis and regulation of carotenoids and anthocyanins, is still lacking.

Mature coffee fruits are oval drupes, approximately 18 mm long and 10–15 mm in diameter, with peels that turn bright red upon ripening, thus earning the nickname “cherry” [12]. Fruit ripening is a complex and delicate physiological process involving pigment biosynthesis, cell division and elongation, accumulation of sugars and organic acids, and the formation of flavor compounds [13]. The dynamic color change in coffee peels from green to yellow to red is the most visible manifestation of this process, influencing not only the visual quality of coffee but also directly correlating with its flavor profile [14,15,16]. Therefore, understanding the molecular mechanisms governing pigment accumulation in coffee peels is essential for optimizing coffee processing and enhancing its quality.

In recent years, high-throughput omics technologies, such as metabolomics and transcriptomics, have provided powerful tools for dissecting plant organ development and pigment synthesis pathways. Studies have successfully elucidated the expression patterns of flavonoids and their key genes in various plant species using techniques like LC-MS/MS and qPCR [17]. However, reports on the combined application of metabolomics and transcriptomics in investigating the coloring mechanism of *Coffea arabica* peels are still limited. In this study, *Coffea arabica* peels at four different maturity stages were utilized, and a joint analysis of metabolomics and transcriptomics was conducted to explore the molecular mechanisms underlying the development and accumulation of peel color. We anticipate that this study will provide novel insights and theoretical foundations for the molecular breeding, quality enhancement, and functional research of *Coffea arabica*.

## 2. Results

### 2.1. Changes in Total Pigment Content Related to the Maturation Process of Arabica Coffee Fruit Peels

**Figure 1:**
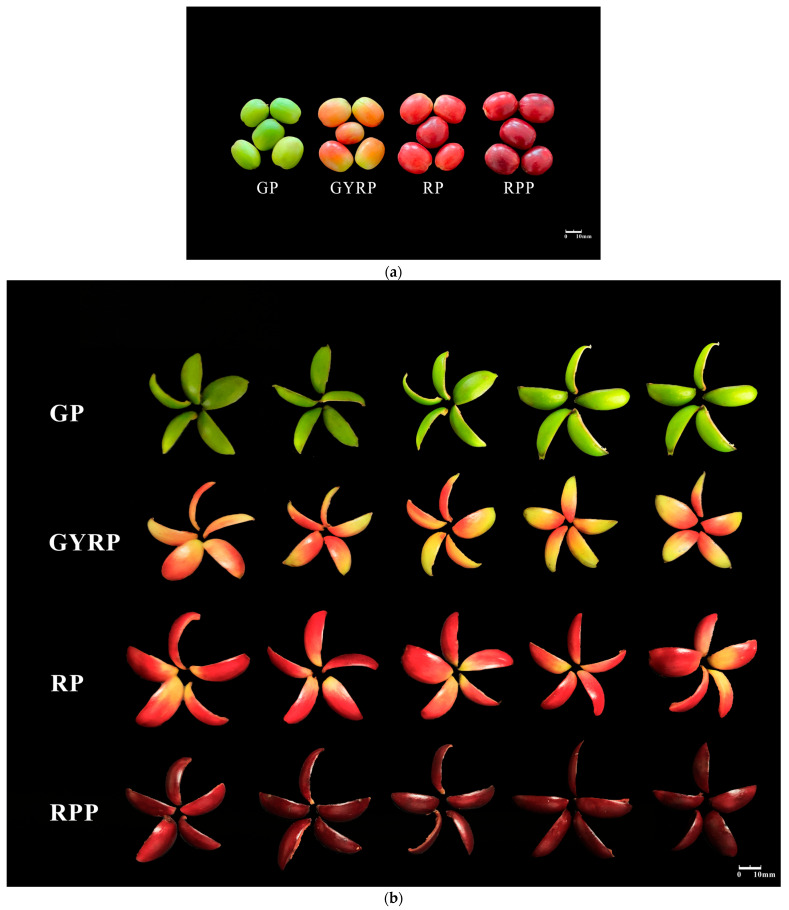

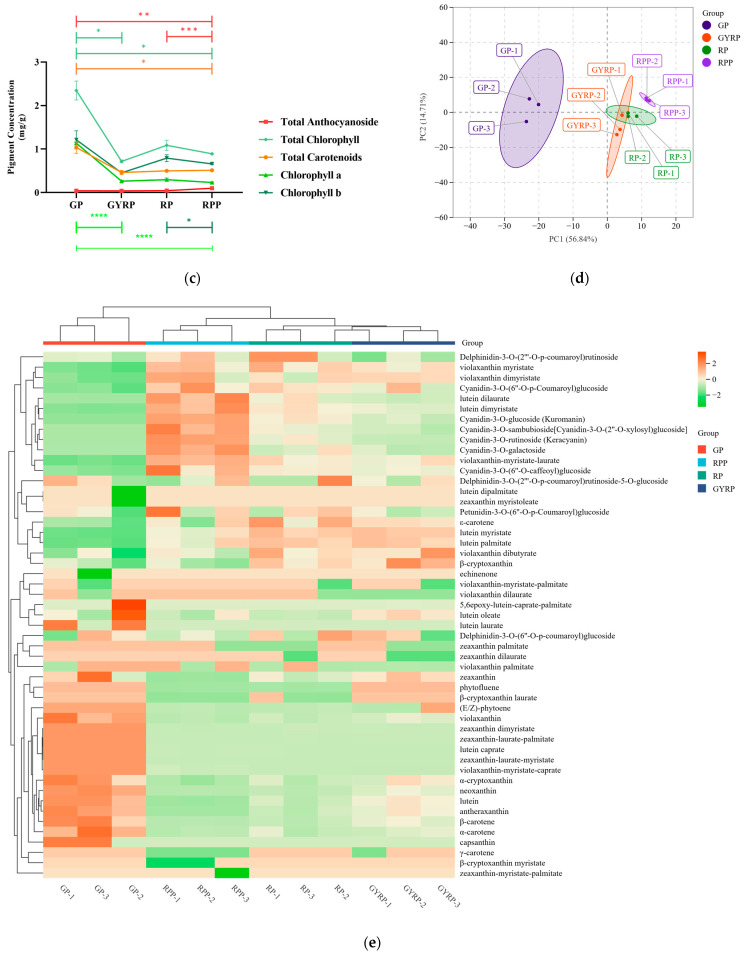
Summary of the maturation process of *Coffea arabica* Fruit peels. (**a**) Images depicting Arabica coffee fruits at four maturity stages: Green (GP, green husk), Green-Yellow Ripe (GYRP, green-yellow husk), Ripe (RP, red husk), and Over-Ripe (RPP, reddish-purple husk) stages. (**b**) Images of Arabica coffee fruits at the Green (GP), Green-Yellow Ripe (GYRP), Ripe (RP), and Over-Ripe (RPP) stages. (**c**) Dynamic variations in the levels (mg/g) of total carotenoids, anthocyanins, total chlorophyll, chlorophyll a, and chlorophyll b in the peels of Arabica coffee during the maturation process, with significant differences highlighted. (**d**) PCA (Principal Component Analysis) plot of metabolites during the maturation process of Arabica coffee. (**e**) Clustering heatmap of metabolites during the maturation process of Arabica coffee. Note: denotes *p* > 0.05, “*” indicates *p* ≤ 0.05, “**” indicates *p* ≤ 0.01, “***” indicates *p* ≤ 0.001, and “****” indicates *p* ≤ 0.0001.

During different developmental stages, the husk color of Arabica coffee gradually transitions from green to purple-red (see Figure 1a,b). To better understand the dynamic changes in pigment content within the peels during this process, this study measured the levels of total chlorophyll, total anthocyanins, and total carotenoids at various maturity stages (Figure 1c). The results indicated that total anthocyanin content increased progressively with fruit maturation; notably, in the RP and RPP stages, total anthocyanin levels in the peels were significantly higher than those in the GP and GYRP stages. Meanwhile, the levels of total chlorophyll and total carotenoids showed opposite trends, with chlorophyll content declining significantly from the GP to the RPP stage. Taken together, these results suggest that the GYRP stage constitutes a critical turning point for the levels of total chlorophyll and total carotenoids, marking a significant shift in husk color from green to purple-red (Figure 1a–c).

### 2.2. Accumulation Differences in Anthocyanin and Carotenoid Metabolism

A principal component analysis (PCA) was employed to analyze metabolomic variations among the sample groups [18]. The PCA results of this study revealed significant metabolomic differences across the four maturity stages (Figure 1d). PC1 (56.84%) and PC2 (14.71%) explained 71.55% of the sample variation, reflecting dynamic shifts in anthocyanin and carotenoid levels at each stage. A hierarchical clustering analysis (HCA) further revealed minor differences between biological replicates, with a high Pearson correlation coefficient of 0.99 for anthocyanin and carotenoid contents (Figure S1), demonstrating good stability and reproducibility of the results. Using UPLC-MS/MS technology, 10 anthocyanins, 6 carotenoids, and 35 xanthophylls were quantified throughout the maturation process (Table S2). An orthogonal partial least squares discriminant analysis (OPLS-DA) demonstrated clear separation between control and treatment groups [19], with high R^2^Y and Q^2^ values for the model (Figure S2), indicating good predictive ability. Several metabolites with high VIP scores were identified as potential biomarkers for differentiating maturity stages.

During the maturation process of the peels from the green to the reddish-purple stage, the accumulation of 10 anthocyanins exhibited a gradual upward trend (Table S3). Notably, Cyanidin-3-O-rutinoside (Keracyanin), Cyanidin-3-O-galactoside, Cyanidin-3-O-sambubioside [Cya-nidin-3-O-(2″O-xylosyl)glucoside], and Cyanidin-3-O-glucoside (Kuromanin) were the main anthocyanins, with Log2 fold change values up to 12.60, indicating substantial accumulation differences. It is worth noting that the Log2 fold change values of anthocyanins did not exceed 2 between the GYRP and RP stages, as well as between the RP and RPP stages, suggesting minor variations. However, when comparing GP with GYRP and GP with RPP, the highest Log2 fold change reached 12.60, highlighting GYRP as a pivotal stage for anthocyanin accumulation, with RPP marking the peak.

To investigate the dynamic changes in carotenoids at different maturity stages, carotenoid levels were quantitatively assessed. A total of 41 carotenoids were identified throughout the maturation process (Table S4), including, α-carotene, β-carotene, phytofluene, γ-carotene, ζ-carotene, ε-carotene, lutein myristate, lutein palmitate, lutein dimyristate, violaxanthin dibutyrate, and violaxanthin myristate. Lutein was the most abundant carotenoid across the four stages, with values of GP (61.15 ± 6.32 μg/g), GYRP (28.96 ± 2.35 μg/g), RP (15.45 ± 2.58 μg/g), and RPP (4.81 ± 0.39 μg/g). Its content was most significant in GP and notably present in GYRP; as the fruit matured, lutein content decreased in the GYRP, RP, and RPP stages. Lutein myristate was notably higher in GYRP (5.28 ± 0.16 μg/g) and RP (5.19 ± 0.35 μg/g), while lutein dimyristate had a significant content in RPP (2.05 ± 0.22 μg/g) (Table S4). These results suggest that different components of carotenoids are crucial in the pigmentation mechanism of husk maturation. In the GP stage, the contents of lutein (61.15 ± 6.32 μg/g), neoxanthin (4.02 ± 0.27 μg/g), β-carotene (3.64 ± 0.58 μg/g), antheraxanthin (2.15 ± 0.25 μg/g), zeaxanthin (2.12 ± 0.83 μg/g), violaxanthin (1.55 ± 0.22 μg/g), α-cryptoxanthin (1.36 ± 0.20 μg/g), and α-carotene (1.24 ± 0.19 μg/g) were highest in GP and gradually decreased through the GYRP, RP, and RPP stages. In particular, α-carotene had the highest content in the GP stage, whereas β-carotene was more abundant in both GP and GYRP stages. These changes may closely correlate with color transitions during husk maturation. In summary, the total carotenoid contents across the stages from GP to RPP were GP (79.54 μg/g), GYRP (49.07 μg/g), RP (34.53 μg/g), and RPP (21.28 μg/g).

### 2.3. Transcriptional Atlas of Coffee Berry Peel during Ripening Process

To further investigate changes in gene expression profiles across the four ripening stages, 12 cDNA libraries were constructed from 12 coffee peel samples representing four consecutive ripening stages with three biological replicates each: GP1, GP2, GP3, GYRP1, GYRP2, GYRP3, RP1, RP2, RP3, RPP1, RPP2, and RPP3. The effective concentrations of these libraries were accurately quantified, with concentrations exceeding 2 nM to ensure high library quality. A transcriptome sequencing analysis was then performed to analyze gene expression profiles. Low-quality reads were removed from each cDNA library to yield high-quality clean reads. A total of 103.32 GB of clean data were generated, with each sample producing at least 6 GB of clean data and a Q30 base percentage exceeding 91% (Table S4). The average total reads for GP, GYRP, RP, and RPP were 62,477,891, 61,633,280, 52,317,812, and 60,097,124, respectively (Table S5). Clean read percentages for GP, GYRP, RP, and RPP were 97.17%, 97.38%, 96.97%, and 96.69%, respectively (Table S5). Furthermore, the error rate, Q20%, Q30%, and GC content were within the ranges of 0.03%, 96.91–97.61%, 91.66–93.39%, and 43.13–44.73%, respectively (Table S5). These metrics indicate high-quality transcriptome sequencing data, supporting the validity of the transcriptome data for downstream analyses. Among the total valid reads, 92.72–93.69% were uniquely aligned with the reference genome (Table S5). A total of 42,141 genes were identified in the coffee peel (Table S7), including 3565 novel genes.

In this study, Diamond [20] was used to annotate the novel genes against sequences in the KEGG, GO, NR, Swiss-Prot, TrEMBL, and KOG databases, with an E-value threshold of 1e−5. Of these, 1844 were successfully annotated in KEGG, 2974 in NR, 1814 in Swiss-Prot, 2956 in TrEMBL, 1541 in KOG, 2353 in GO, and 1661 in Pfam. The successful annotation of these novel genes enriched the genomic dataset for coffee peel ripening (Table S8).

### 2.4. Comparative Analysis of Differentially Expressed Genes

**Figure 2:**
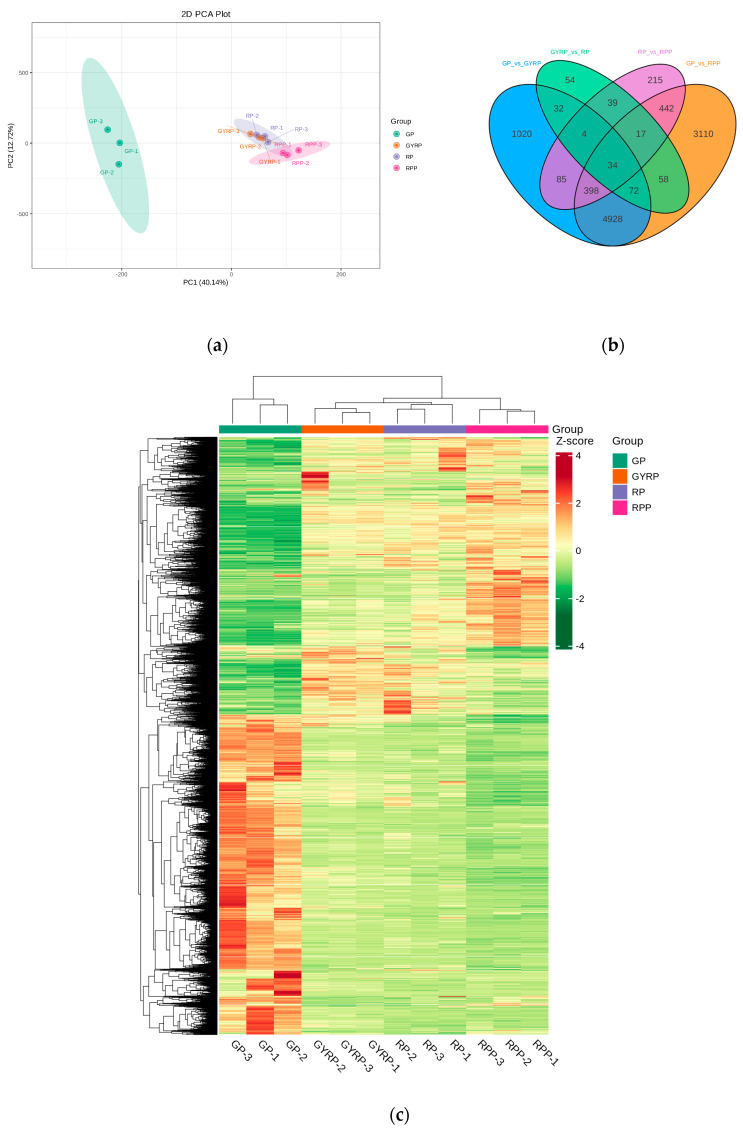
Gene expression profiles during the ripening process of coffee beans from Arabica species. (**a**) Principal component analysis (PCA) of gene expression across ripening stages. The stages include: Green Peel (GP), Green−Yellow−Red Peel (GYRP), Red Peel (RP), and Red−Purple Peel (RPP). (**b**) Venn diagrams of differentially expressed genes (DEGs) during ripening. The diagrams compare DEGs between GP vs. GYRP, GYRP vs. RP, RP vs. RPP, and GP vs. RPP. Overlapping areas indicate shared DEGs between comparison groups, whereas non−overlapping areas represent unique DEGs specific to each comparison. (**c**) Heatmap of clustered DEGs. The horizontal axis shows sample names and the results of hierarchical clustering, while the vertical axis displays DEGs and their corresponding clustering results. Red indicates high expression, and green indicates low expression.

A multivariate statistical analysis using principal component analysis (PCA) was performed on the sample groups (Figure 2a). The results revealed that all biological replicates clustered together, demonstrating high sequencing data reliability. The first principal component (PC1) explained 40.14% of the variance, representing differences among samples during the ripening process (Figure 2a). Similar to the metabolome analysis, a clear separation was observed between the colored samples, indicating gene expression variations during Arabica coffee bean ripening. The criteria for selecting differentially expressed genes in this study were |log2Fold Change| ≥ 1 and FDR < 0.05. An analysis of the Venn diagram for differentially expressed genes highlighted distinct differences across ripening comparisons, with the highest number of unique genes found in GP vs. RPP (3110 genes) and the lowest in GYRP vs. RP (54 genes), while 34 genes were common to all four comparisons. Hence, it was initially determined that the significant difference between GP and RPP was linked to ripening stages.

A hierarchical clustering analysis was conducted on the combined set of differentially expressed genes across comparisons, with data standardized using Z−score normalization. A clustering heatmap was generated for each differential group (Figure 2c), revealing distinct expression profiles between GP and the other stages. The dendrogram further showed distinct expression patterns for GP compared to the other stages, which was consistent with the metabolite clustering (Figure 2c) and Venn diagram results. After standardizing the FPKM values of all differential genes using the scale() function in R, a k-means clustering analysis was conducted, revealing similar expression trends for genes within clusters across ripening stages, indicating potential functional similarities (Figure S4).

**Figure 3:**
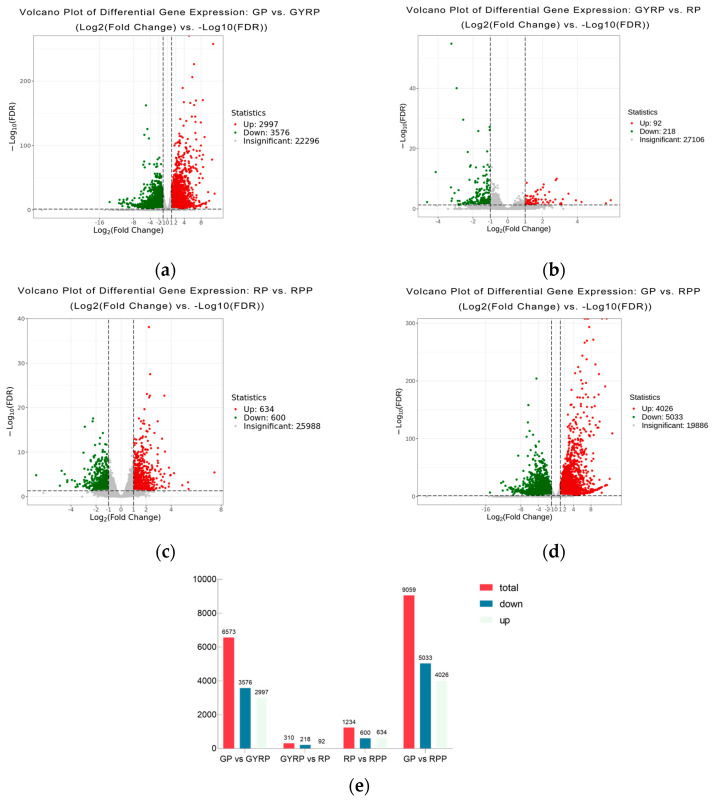
Overview of differentially expressed genes (DEGs): (**a**–**d**) present volcano plots for DEGs in the comparisons of GP vs. GYRP, GYRP vs. RP, RP vs. RPP, and GP vs. RPP, respectively. The horizontal axis represents the fold change in gene expression, while the vertical axis indicates the significance level of the DEGs. Red dots signify upregulated DEGs, green dots represent downregulated DEGs, and gray dots denote genes with no significant differential expression. (**e**) Number of DEGs in pairwise comparisons during the ripening process of Arabica coffee beans, including: Green Peel (GP), Green−Yellow Peel (GYRP), Red Peel (RP), and Red−Purple Peel (RPP). The total number is a summation of both downregulated and upregulated genes.

Across the GP, GYRP, RP, and RPP comparisons (Figure 3), a total of 6573 genes were differentially expressed in GP vs. GYRP (3576 downregulated and 2997 upregulated), 9059 genes in GP vs. RPP (5033 downregulated and 4026 upregulated), 310 genes in GYRP vs. RP (218 downregulated and 92 upregulated), and 1234 genes in RP vs. RPP (600 downregulated and 634 upregulated). Overall, 11,165 genes were differentially expressed across the four pairwise comparisons. Enriched pathways were identified for the differentially expressed genes during the ripening process and fruit skin coloration in Arabica coffee, based on a *p*-value of 0.05 in GP vs. GYRP, GYRP vs. RP, RP vs. RPP, and GP vs. RPP, resulting in a total of 55 enriched pathways (Table S7). These enriched pathways indicate that the ripening process and fruit skin coloration in Arabica coffee involve not only flavonoid, anthocyanin, and carotenoid biosynthesis but also quality-related changes, such as fructose and mannose metabolism, and starch and sucrose metabolism.

### 2.5. Dynamic Changes in Enzymes Related to Anthocyanin and Carotenoid Biosynthesis

**Figure 4:**
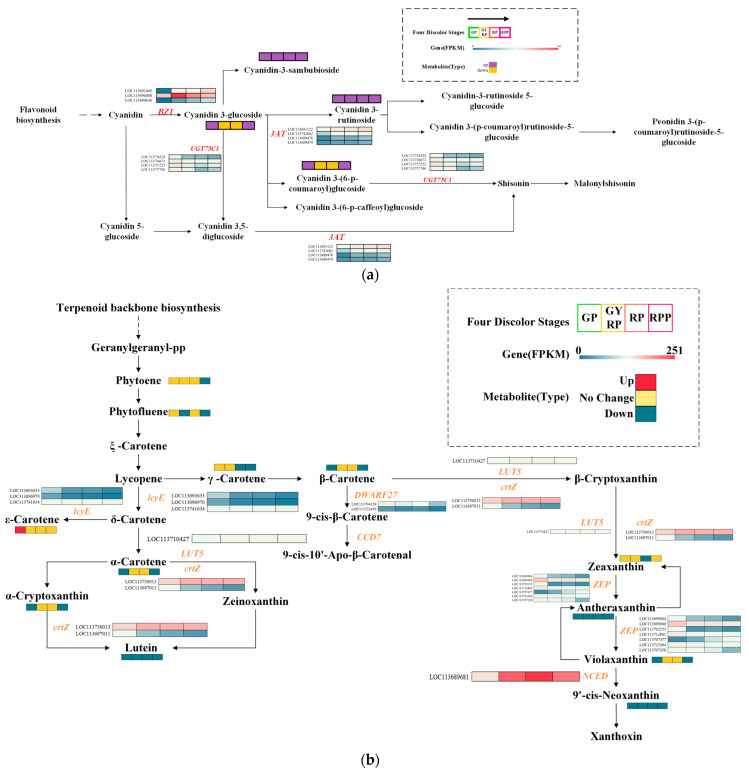
Biosynthesis of anthocyanins and carotenoids during the ripening process of Arabica coffee peels. (**a**) Schematic of anthocyanin biosynthesis showing differentially expressed genes (FPKM) and metabolites involved at four stages, indicated by distinct colors. Key enzymes involved are BZ1 (anthocyanidin 3-O-glucosyltransferase [EC: 2.4.1.115]), 3AT (anthocyanidin 3-O-glucoside 6″-O-acyltransferase [EC:2.3.1.215]), and UGT75C1 (anthocyanidin 3-O-glucoside 5-O-glucosyltransferase [EC:2.4.1.298]). (**b**) Schematic of carotenoid biosynthesis showing differentially expressed genes (FPKM) and metabolites involved at four stages, indicated by distinct colors. Key enzymes involved are lcyE (lycopene epsi-lon-cyclase [EC:5.5.1.18]), LUT5 (beta-ring hydroxylase [EC:1.14.-.-]), crtZ (be-ta-carotene 3-hydroxylase [EC:1.14.15.24]), DWARF27 (beta-carotene isomerase [EC:5.2.1.14]), CCD7 (9-cis-beta-carotene 9′,10′-cleaving dioxygenase [EC:1.13.11.68]), ZEP (zeaxanthin epoxidase [EC:1.14.15.21]), NCED (9-cis-epoxycarotenoid dioxygenase [EC:1.13.11.51]), ABA2 (xanthoxin dehydro-genase [EC:1.1.1.288]), AAO3 (abscisic-aldehyde oxidase [EC:1.2.3.14]), CYP707A ((+)-abscisic acid 8′-hydroxylase [EC:1.14.14.137]), and AOG (abscisate be-ta-glucosyltransferase [EC:2.4.1.263]). Note: the enzymes and their corresponding EC numbers are provided to aid in understanding the biosynthetic pathways. The differentially expressed genes (FPKM) and metabolites (Type) at each stage are indicated by colors that represent their expression levels and types during the ripening of Arabica coffee bean peels.

To delve deeper into the regulatory mechanisms of anthocyanin and carotenoid biosynthesis, the study annotated the expressed genes and differentially accumulated metabolites (Log2|Fold change| > 1 and VIP ≥ 1) related to anthocyanin and carotenoid biosynthetic pathways in the KEGG Database, reconstructing the biosynthetic pathways of anthocyanins and carotenoids. The results identified three key enzymes involved in anthocyanin biosynthesis: anthocyanin 3-O-glucosyltransferase (BZ1), anthocyanin-3-O-glucoside-6″-O-acyltransferase (3AT), and anthocya-nin-3-O-glucoside-5-O-glucosyltransferase (UGT75C1) (Figure 4a). Compared to GP, the expression levels of these enzyme genes increased between 2-fold and 9-fold in the GYRP, RP, and RPP stages. Specifically, cyanidin 3-rutinoside and cya-nidin-3-sambubioside were significantly elevated in GP vs. GYRP, GYRP vs. RP, and RP vs. RPP comparisons, while cyanidin 3-glucoside and cyanidin 3-(6-p-caffeoyl)glucoside were upregulated in GP vs. GYRP and GP vs. RPP but downregulated in GYRP vs. RP and RP vs. RPP. The expression trends in these genes followed RPP > RP > GYRP > GP, suggesting restricted anthocyanin biosynthesis during fruit development in the GP stage compared to GYRP, RP, or RPP.

In carotenoid biosynthesis, 32 regulatory enzyme genes were identified (Figure 4b). Among them, the high expression of the crtZ enzyme (LOC11370013) was associated with lutein downregulation across GP vs. GYRP, GYRP vs. RP, RP vs. RPP, and GP vs. RPP comparisons. Additionally, genes such as lcyE (LOC113693633, LOC113696970), crtZ (LOC113697011), DWARF27 (LOC113704159), ZEP (LOC113699064, LOC113702153, LOC113712881), AAO3 (LOC113725547), and CYP707 (LOC113731421, LOC113698442, LOC113722535) exhibited higher expression levels (FPKM) in GP compared to GYRP, RP, or RPP stages. This suggests higher enzyme activity during carotenoid biosynthesis in the GP stage, contributing to yellow pigmentation. In the GYRP, RP, and RPP stages, the high expression of NCED and ABA2 may primarily drive yellow pigmentation.

### 2.6. WGCNA-Based Network Analysis

To identify co-expressed gene modules and investigate the relationships between gene networks and the biosynthesis of anthocyanins and carotenoids, this study conducted a weighted gene co-expression network analysis (WGCNA), systematically dissecting the dynamic characteristics of total expressed genes and pigment-related differential metabolites during the ripening process. The analysis included 42,141 genes and 28 differential metabolites. By measuring the relationship between correlation coefficients and average connectivity across different thresholds (power values ranging from 1 to 20), the analysis determined that a power value of 20 balanced correlation coefficients and average connectivity well (Figure S5). Ultimately, 14 modules were identified in the clustering dendrogram (Figure S6), each containing a different number of genes. The turquoise module had the most genes (13,589), while the salmon module had the fewest (96), averaging 2107 genes per module.

**Figure 5:**
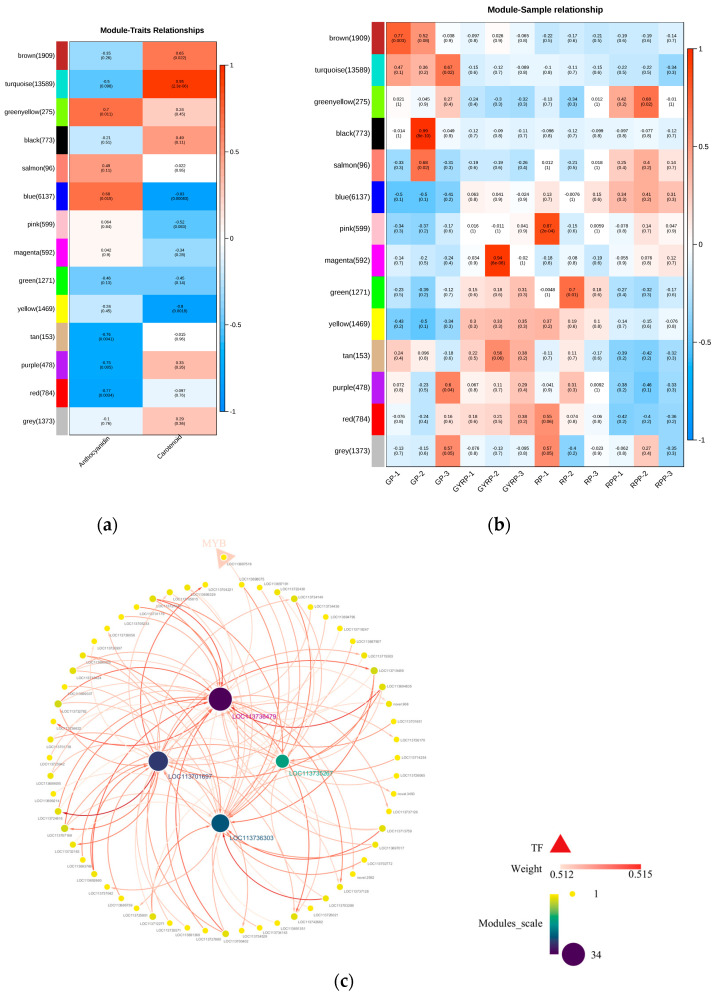

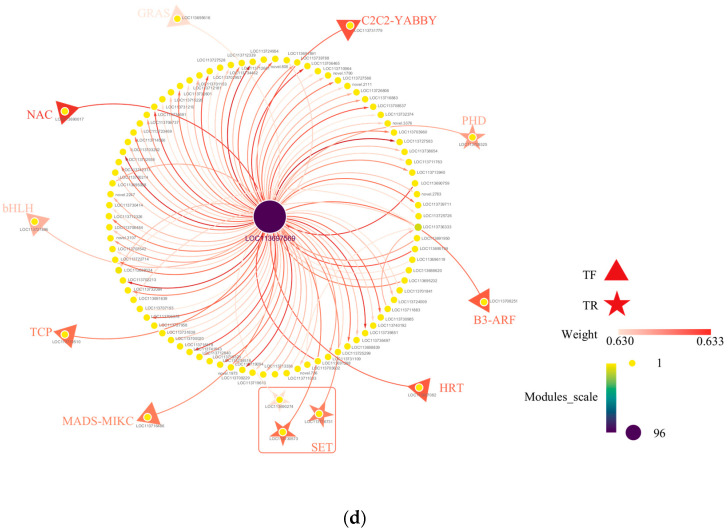
Weighted gene co−expression network analysis (WGCNA) of stable and highly expressed genes during the four pigmentation stages of Arabica coffee bean peels. (**a**) Shows the heatmap depicting correlations between total anthocyanin and carotenoid contents and the gene modules. (**b**) Illustrates the heatmap representing correlations between samples and gene modules. (**c**) Displays the interaction network of core genes within the blue module, whereas (**d**) presents the network for the turquoise module. Note: in (**c**,**d**), arrows indicate interactions from the ‘fromNode’ to the ‘toNode’. Connection strength is represented by ‘weight’ (edge weight in the adjacency matrix), indicating the intensity between two nodes or genes.

A Pearson correlation analysis was performed between the genes in each module and total anthocyanin content, total carotenoid content, and sample metabolites (Figure 5a,b). The results revealed that five modules were positively correlated with total anthocyanin content, and eight modules were negatively correlated. Similarly, six modules were positively correlated with total carotenoid content, while seven modules were negatively correlated. The green-yellow module showed the highest positive correlation with total anthocyanin content, while the blue module exhibited the highest negative correlation. Conversely, the turquoise module demonstrated the highest positive correlation with total carotenoid content, whereas the blue module had the highest negative correlation. Specifically, GP showed positive correlations with four modules and negative correlations with six modules. In the sample groupings, GYRP showed positive correlations with six modules and negative correlations with six modules; RP was positively correlated with four modules and negatively correlated with seven modules; and RPP was positively correlated with five modules and negatively correlated with nine modules. Notably, the turquoise module was exclusively positively correlated with GP. The blue module genes exhibited a trend consistent with the change in anthocyanin content during the ripening process, showing a gradual increase in correlation from GP to RPP. In the heatmap depicting correlations between metabolites and module genes, a high correlation between blue module genes and anthocyanin metabolites was observed (Figure 5b), while strong correlations with carotenoids were primarily concentrated in the turquoise module. The correlation of yellow module genes gradually decreased across the GYRP, RP, and RPP stages.

Based on the top 10 hub genes and their 100 most weighted interacting gene pairs in the blue and turquoise modules, interaction networks were constructed for both modules (Figure 5c,d). The results indicated that the top 4 genes with the highest interaction frequencies in the blue module were gene-LOC113736479 (34 times), gene-LOC113701697 (27 times), gene-LOC113736303 (24 times), and gene-LOC113735267 (15 times), among which gene-LOC113736479 is likely pivotal in anthocyanin biosynthesis. In the turquoise module, gene-LOC113697569 had an interaction frequency of up to 96 times, indicating its significant role in carotenoid biosynthesis.

Within the blue module, the MYB transcription factor (gene-LOC113697518), although weakly correlated with gene-LOC113736479 (weight = 0.512522599), showed 34 interactions. In the turquoise module, the top 100 interacting gene pairs included several transcription factors (TFs) and transcriptional regulators (TRs), such as the NAC transcription factor (gene-LOC113690017), GRAS transcription factor (gene-LOC113695616), C2C2-YABBY transcription factor (gene-LOC113731779), and SET transcriptional regulator (gene-LOC113730573). The NAC transcription factor (gene-LOC113690017) was most correlated with gene-LOC113697569 (weight = 0.632559756), followed by the C2C2-YABBY transcription factor (gene-LOC113731779, weight = 0.632209587). These results suggest that gene-LOC113736479 is crucial in regulating anthocyanin biosynthesis, while gene-LOC113697569 is key for carotenoid biosynthesis.

### 2.7. Validation of RNA-Seq Data by qPCR

To validate the RNA-Seq data and assess the expression profiles of genes related to anthocyanin biosynthesis, six structural genes involved in anthocyanin and carotenoid synthesis, along with four additional genes from the MYB, bHLH, and NAC families, were randomly selected for quantitative PCR (qPCR) detection. The relative expression levels of these genes showed variation during maturation, with expression patterns consistent with the RNA-Seq data (Figure S7). These findings further validated the reliability of the RNA-Seq results.

## 3. Discussion

Natural pigments in fruit peels play crucial roles in plant growth, development, photosynthesis, attraction of pollinators, seed dispersal, and resistance against biotic and abiotic stresses. Among the primary pigments determining fruit peel color, carotenoids exhibit red, yellow, and orange hues, flavonoids produce yellow, and anthocyanins display red, purple, and blue colors. These pigments possess potent anti-oxidant properties and offer various health benefits, such as delaying aging, repairing the nervous system, combating atherosclerosis, and exhibiting anticancer and anti-inflammatory effects [21,22,23]. Consequently, natural pigments have been a focal point of research. Previous studies have shown that the color of yellow-skinned varieties of *Coffea arabica* is attributed to carotenoids, while the colors of orange- and red-skinned varieties are determined by carotenoids and varying levels of anthocyanins, respectively [6,24,25]. However, earlier research on coffee fruit peel pigments primarily focused on a limited number of metabolites, including anthocyanins, carotenoids, and phenols, without comprehensively elucidating the pigmentation mechanism of natural pigments in the peel. This study, through targeted metabolomic and transcriptomic integrative analysis of anthocyanins and carotenoids in *Coffea arabica* fruit peels at four maturity stages (GP, GYRP, RP, and RPP, see Figure 1), delineates the mechanism of peel pigmentation during ripening and provides a comprehensive overview of the dynamic changes in metabolites, gene expression, and transcription factors involved in the ripening process of *Coffea arabica*. Initially, by analyzing the dynamic changes in pigment content in *Coffea arabica* fruit peels, GYRP was identified as a critical juncture for pigment transformation (see Figure 2). This was further validated through metabolomic and transcriptomic analyses, revealing significant differences in metabolite and gene expression patterns between GYRP, RP, and RPP compared to GP (see Figure 1 and Figure 2a,c).

In the detection of anthocyanins and carotenoids, a total of 51 metabolites were identified (Table S1), providing a comprehensive metabolic profile of anthocyanins and carotenoids related to color changes during the ripening of *Coffea arabica*. Anthocyanins are the most significant flavonoid pigments in plants, particularly prominent in fruits such as plums, berries, and grapes, contributing to red, blue, and purple hues [26]. Previous research has also emphasized the role of anthocyanins in the pigmentation of *Coffea arabica* fruit peels [24], but earlier studies did not delve into the dynamic changes and underlying causes of anthocyanin accumulation during coffee ripening. In this study, we analyzed the ripening process of coffee fruit peels to uncover the dynamic changes in anthocyanins (see Table S2). A total of ten anthocyanins were detected. Results from the green peel stage to the red-purple peel stage indicated that four anthocyanins—Cyanidin-3-O-rutinoside (Keracyanin), Cyanidin-3-O-galactoside, Cyanidin-3-O-sambubioside [Cya-nidin-3-O-(2″-O-xylosyl)glucoside], and Cyanidin-3-O-glucoside (Kuromanin)—exhibited significantly differential accumulation with notably higher levels than other groups and increased progressively as the peel turned red. These four anthocyanins may be key factors in the accumulation of red pigments during the ripening of coffee fruit peels. Notably, recent studies have confirmed that these four anthocyanins can induce reddening in plant tissues [27,28]. Furthermore, an analysis of the biosynthetic pathway of anthocyanins suggested that the changes in these four anthocyanins might be regulated by BZ1 enzyme genes (LOC11369144, LOC113694480, LOC113694636) and 3AT enzyme genes (LOC113691122, LOC113743602, LOC113689476, LOC113689479). Previous research has also indicated that BZ1, 3AT, and UGT75C1 enzyme genes play major roles in anthocyanin synthesis [29,30,31].

Thylakoid membranes and plastoglobuli in chloroplasts efficiently sequester and store carotenoids in plastids, leading to their high accumulation in green tissues and influencing the nutritional value of plant tissues [32]. This study examined the carotenoid composition in four fruit samples (see Table S4), identifying 41 carotenoid components. The total carotenoid content was highest in green peel (GP) (79.54 μg/g), followed by green-yellow-red peel (GYRP) (49.07 μg/g), red peel (RP) (34.53 μg/g), and red-purple peel (RPP) (21.28 μg/g). These findings suggest that carotenoids are primary contributors to pigmentation in GP and GYRP, whereas anthocyanins dominate in RP and RPP. During *Coffea arabica* ripening, carotenoid biosynthesis is regulated by enzymes such as lcyE, crtZ, DWARF27, NCED, ZEP, ABA2, AAO3, and CYP707. Previous studies have shown that xanthophylls and β-carotenoids are the predominant carotenoids, primarily regulated by enzyme genes [33]. Furthermore, crtZ, ZEP, ABA2, and NCED are critical genes in carotenoid biosynthesis and accumulation [34,35]. This study supports these findings and provides new insights into the molecular mechanisms of anthocyanin and carotenoid synthesis in *Coffea arabica*. The biosynthesis of anthocyanins is regulated by transcription factors such as MYB, bHLH, WRKY, bZIP, and NAC [36], while the regulatory mechanism of the carotenoid pathway remains contentious, potentially involving MYB, bHLH, or AP2/ERF-ERF [37,38].

A co-expression network of 42,141 genes and 28 differential metabolites was developed through a weighted gene co-expression network analysis (WGCNA). Results showed that anthocyanin and carotenoid metabolites were strongly associated with the blue and turquoise modules (see Figure 5). The ten hub genes identified via WGCNA (see Table S9) revealed that the core gene in the module is gene-LOC113736479 (module membership of 34), which interacts strongly with MYB TF (weight = 0.512522599), indicating that MYB TF (gene-LOC113697518) is a primary regulator of anthocyanin biosynthesis during *Coffea arabica* peel ripening. Previous research has suggested that transcription factor families such as MYB, bHLH, and NAC are involved in regulating anthocyanin synthesis [37]. In the turquoise module, highly correlated with carotenoids, interactions between hub genes and transcription factors such as GRAS, C2C2-YABBY, B3-ARF, HRT, and MADS-MIKC were also identified, validating previous research and revealing significant interactions between SET and PHD TR within the turquoise module. SET family proteins play essential roles in diverse organisms, particularly in lysine methylation and chromatin regulation [39]. These proteins influence gene expression by modulating the methylation status of histones, thus impacting biological growth, development, and immune responses. Previous studies have shown that PTM (PHD-type transcription factors) can sense chloroplast signals and respond to environmental changes and metabolic demands by modulating the expression of specific genes (e.g., ABI4) in the nucleus, while carotenoids are integral components of photosynthesis and are influenced by chloroplast function [40]. This further enriches our understanding of transcriptional regulatory mechanisms in *Coffea arabica* ripening. Future research could clarify the roles of these highly interactive transcription factors and SET and PHD proteins in plant pigment metabolism through validation of their regulatory relationships with key genes in anthocyanin and carotenoid biosynthetic pathways.

Mature fruits offer economic advantages through primary and secondary processing, attributed to their high quality and nutritional value. This study demonstrates that changes in the peel color of *Coffea arabica* are linked to changes in metabolites and differential gene expression. Although these genes are not directly related to peel pigmentation, they are significantly associated with fruit quality and nutrition. For instance, glycerophospholipid metabolites showed significant enrichment in both GP and RPP (see Table S7). This study also revealed enriched pathways during fruit ripening through a KEGG pathway enrichment analysis of differential metabolites and differentially expressed genes, potentially highlighting links between fruit color changes and quality and nutritional alterations. For example, in the KEGG analysis of GP and GYRP, differentially accumulated metabolites and expressed genes were significantly enriched in fructose and mannose metabolism. Related studies have shown that fructose, as a signaling molecule, participates in many metabolic and developmental processes and acts as a central hub for growth, nutrition, and stress signaling in plants [41,42].

It has long been hypothesized that the transition from fruit development to ripening is guided by non-systemic developmental signals, allowing plants to bear fruits of different maturities simultaneously [43]. The uneven ripening of coffee fruits results in the simultaneous appearance of differently colored fruits, complicating the harvesting process [15]. Additionally, studies have shown that classifying coffee fruits based on their ripening stages aids in developing selective harvesting methods in engineering fields such as robotics, machine vision, and vibration systems, including methods for developing color charts of coffee fruit ripeness using digital image processing and colorimetry [44]. In *Coffea arabica*, the color change from green to red peel represents a key breeding trait. In the future, new cultivation strategies can be developed by regulating and further analyzing these key enzyme genes and transcription factors, facilitating the advancement of intelligent identification and harvesting, and the development of high-quality, high-value, and nutritious coffee byproducts.

## 4. Materials and Methods

### 4.1. Plant Materials

In this study, fresh fruit peels of the *Catimor* Population Thailand (*Catimor* PT) cultivar of the *Catimor* coffee lineage were utilized as plant material. The samples were grown in the Coffee Germplasm Resource Garden of the Tropical Crops College at Yunnan Agricultural University (101°04′30.26″ E, 22°45′08.52″ N), situated at an altitude of 1442.47 m. The region experiences annual average temperatures ranging from 15 to 20.3 °C and a humidity level of 77.9%, with long annual sunshine duration, a frost-free period exceeding 315 days, and annual rainfall between 1100 and 2780 mm. After harvesting, the samples were pooled according to maturity stage. After portioning, samples were flash-frozen with liquid nitrogen and stored in dry ice-filled sampling boxes before shipment for analysis. The portions for physicochemical analysis were immediately returned to the laboratory for the assessment of husk color and related physicochemical indicators. The study comprised four maturity treatment groups, each with three biological replicates, yielding a total of 12 samples. These were used for three analytical approaches (metabolomics, transcriptomics, and physicochemical analysis), resulting in a total of 36 samples. The fresh peels were then stored at −0 °C.

### 4.2. Determination of Total Anthocyanins, Carotenoids, and Chlorophyll Content

The determination of total anthocyanin content, total carotenoid content, and total chlorophyll content was performed according to the methods described by Wang et al. [36].

### 4.3. Sample Preparation and Metabolite Extraction

The metabolomic analysis of anthocyanins and carotenoids was conducted by Wuhan Metware Biotechnology Co., Ltd. (Wuhan, China) using UPLC-MS/MS. The ultra-performance liquid chromatography system (UPLC) was ExionLC™ AD (SCIEX, Framingham, MA, USA), and the tandem mass spectrometry system (MS/MS) was 4500 QTRAP (Applied Biosystems, Foster City, CA, USA). Frozen plant tissue samples were ground into powder using an MM 400 mill (Retsch GmbH & Co. KG, Haan, North Rhine-Westphalia, Germany) at 30 Hz for 1.5 min. For anthocyanin metabolite detection, 50 mg of the sample was dissolved in 1200 μL of pre-cooled (−20 °C) 70% methanol water with an internal standard extraction solution (for samples < 50 mg, the extraction agent was proportionally adjusted to 1200 μL per 50 mg). The solution was vortexed every 30 min for 30 s, repeated six times. Centrifugation at 13,523 g for 3 min followed by filtration through a 0.22 μm filter membrane. The extract was then analyzed using an Applied Biosystems 4500 QTRAP LC−MS/MS system. The liquid chromatography conditions were as follows: chromatographic column: Agilent SB−C18 1.8 μm, 2.1 mm × 100 mm; mobile phase A: ultrapure water with 0.1% formic acid; mobile phase B: acetonitrile with 0.1% formic acid. For carotenoid metabolite detection, 50 mg of ground sample was extracted with 0.5 mL of hexane/acetone/ethanol (1:1:1, *v*/*v*/*v*) with 0.01% BHT. After vortexing at room temperature for 20 min, the solution was centrifuged at 13,523× *g* for 5 min at 4 °C, and the supernatant was collected. The extraction was repeated, and supernatants were combined. The resulting extract was concentrated, redissolved in 100 μL of dichloromethane, filtered through a 0.22 μm filter membrane, and analyzed using a QTRAP^®^ 6500 + LC-MS/MS system(SCIEX, Frisco, Texas, USA). The chromatographic column was YMC C30 (3 μm, 100 mm × 2.0 mm i.d.); mobile phase A: methanol/acetonitrile (1:3, *v*/*v*) with 0.01% BHT and 0.1% formic acid; mobile phase B: methyl tert-butyl ether with 0.01% BHT.

### 4.4. Metabolomic Data Analysis

Based on the MWDB (Metware Database) constructed from standard compounds, substances were qualitatively analyzed using secondary spectral data. Isotope signals, repeated signals containing K^+^, Na^+^, and NH_4_^+^ ions, and repeated signals of fragment ions from larger molecular weight substances were excluded during analysis. A metabolite data analysis was conducted with Analyst 1.6.3 (SCIEX, Framingham, MA, USA). Unsupervised multidimensional statistical analysis methods and a orthogonal partial least squares discriminant analysis (OPLS-DA) were applied to enhance metabolomic differences between sample pairs [18,45]. Unsupervised PCA (principal component analysis) was conducted with the pre-comp function in R v4.3.1 (R Foundation for Statistical Computing, Vienna, Austria). For two-group analysis, differential metabolites were identified using VIP (≥1) and absolute Log2FC (≥1.0). HCA (hierarchical clustering analysis) results for samples and metabolites were displayed as heatmaps with dendrograms, while Pearson correlation coefficients (PCC) between samples were calculated using the core function in R (v4.3.1) and presented as heatmaps. Both HCA and PCC were performed with the ComplexHeatmap package in R.

### 4.5. RNA Extraction, Quantification, and Sequencing

Twelve libraries representing four peel samples, and three replicates were prepared for transcriptome sequencing. RNA extraction, quantification, and transcriptome sequencing followed rigorous quality control, as detailed by Chen et al. [46]. After passing library quality inspection, different libraries were pooled based on effective concentration and target data volume and then sequenced using Illumina technology to generate 150 bp paired-end reads.

### 4.6. Transcriptome Data Analysis

Raw data were filtered using fastp, primarily to remove reads with adapters. Reads were discarded when the N content in any sequencing read exceeded 10% of the base count or when the number of low-quality (Q ≤ 20) bases exceeded 50% of the read’s base count. All subsequent analyses used clean reads. The reference genome (GCF_003713225.1_Cara_1.0_genomic.fna.gz) and its annotation file (GCF_003713225.1_Cara_1.0_genomic.gff.gz) were obtained from the NCBI database (https://www.ncbi.nlm.nih.gov/genome/?term=Coffea+arabica+genome accessed on 6 February 2023). An index was constructed using HISAT2 [47], and clean reads were aligned to the reference genome. New gene prediction was performed using StringTie [48], applying network flow algorithms and optional de novo transcript assembly. The sequences of new genes were extracted from the genome, and the new genes were annotated by comparing them with sequences in the Kyoto Encyclopedia of Genes and Genomes (KEGG), Gene Ontology (GO), NCBI Non-Redundant Protein Database (NR), Swiss-Prot Protein Database (Swiss-Prot), Translated EMBL Nucleotide Sequence Database (TrEMBL), and EuKaryotic Orthologous Groups (KOG) databases using diamond [20] with an E-value threshold of 1e−5. Gene alignment was calculated using featureCounts [49], and the FPKM (Fragments Per Kilobase of transcript per Million fragments mapped) was calculated for each gene based on gene length. FPKM is widely used for estimating gene expression levels. Differential expression analysis between groups was performed using DESeq2 [50,51], with *p*-values adjusted by the Benjamini and Hochberg method. The corrected *p*-values and log2fold changes served as thresholds for significant differential expression. Enrichment analysis was per-formed using pathways for KEGG and GO terms for GO. Weighted gene co-expression network analysis was conducted using WGCNA software version 1.69 (R Foundation for Statistical Computing, Vienna, Austria), setting mergeCutHeight to 0.25.

### 4.7. Validation of RNA-Seq by Quantitative PCR

MonScript™ RTIII All-in-One Mix with dsDNase (Monad, Suzhou, China) was used for reverse transcription of RNA to generate cDNA. Primers were designed as listed in Supplementary Table S1. The QuantiNova SYBR Green PCR Kit (QIAGEN, Hilden, Germany) was used along with the ABI 7500 fluorescent quantitative PCR instrument (Applied Biosystems, Foster City, CA, USA) for qPCR experiments. All qPCR experiments were performed with three technical replicates and three biological replicates to ensure result reliability and stability. GAPDH and Actin were used as reference genes, and relative expression levels were determined by the 2^−ΔΔCt method [52].

### 4.8. Data Analysis

Data were initially organized using Excel 2016 (Microsoft Corporation, Redmond, WA, USA) and then further analyzed with SPSS 26.0 (IBM, Armonk, NY, USA) software. Before performing a one-way analysis of variance (ANOVA), the normality and homoscedasticity of the data were tested to confirm ANOVA applicability. For changes in pigment content during ripening, multiple comparisons were conducted using the Tukey method (mean ± SD), and significant differences in total pigment content between different time points were determined (significance level set at *p <* 0.05). For differences in the accumulation of various metabolites during carotenoid metabolism, the Duncan method was used for multiple comparisons (mean ± SE). The Duncan method often provides more robust statistical inferences for data with non-normal distribution or heteroscedasticity. Additionally, R v4.3.1 (R Foundation for Statistical Computing, Vienna, Austria) and GraphPad Prism 10.0 (GraphPad Software, San Diego, CA, USA) software were employed for graphing. All analyses were based on data from three biological replicates to ensure experimental result re-liability and stability.

## 5. Conclusions

This study examined the molecular mechanisms underlying pigmentation during *Coffea arabica* ripening through metabolomics and transcriptomics approaches, identifying GYRP as a critical stage for peel color transition. Specifically, the study found that the accumulation of carotenoids and anthocyanins, with a notable increase in four key anthocyanins—Cyanidin-3-O-rutinoside (Keracyanin), Cyanidin-3-O-galactoside, Cyanidin-3-O-sambubioside [Cyanidin-3-O-(2″-O-xylosyl)glucoside], and Cyanidin-3-O-glucoside (Kuromanin)—is strongly associated with red pigmentation in *Coffea arabica* peel, while yellow pigmentation is primarily determined by carotenoids, as expected, given that anthocyanins are the primary contributors to red coloration. Several key enzyme genes, including 3AT, BZ1, and lcyE, involved in anthocyanin and carotenoid biosynthesis in *Coffea arabica* peel were identified. Furthermore, core regulatory genes and associated transcription factors, such as MYB, bHLH, and NAC transcription factors, were uncovered via a WGCNA. These findings provide valuable insights into the genetic regulation of anthocyanin biosynthesis in *Coffea arabica* peel. The data presented could guide future efforts in genetic engineering, such as enhancing pigment production through targeted manipulation of biosynthetic pathways.

## Data Availability

No data were used for the research described in this article. The datasets presented in this study can be found in online repositories.

## Supplementary Materials

The following supporting information can be downloaded at: https://www.mdpi.com/article/10.3390/ijms251910754/s1.
