# Supplementary figures and images for: Integrated Metabolomics and Transcriptomics Analyses Reveal the Regulatory Mechanisms of Anthocyanin and Carotenoid Accumulation in the Peel of Coffea arabica
Source: Int J Mol Sci. 2024 Oct 6;25(19):10754. doi: 10.3390/ijms251910754 (PMC11477210; doi:10.3390/ijms251910754)

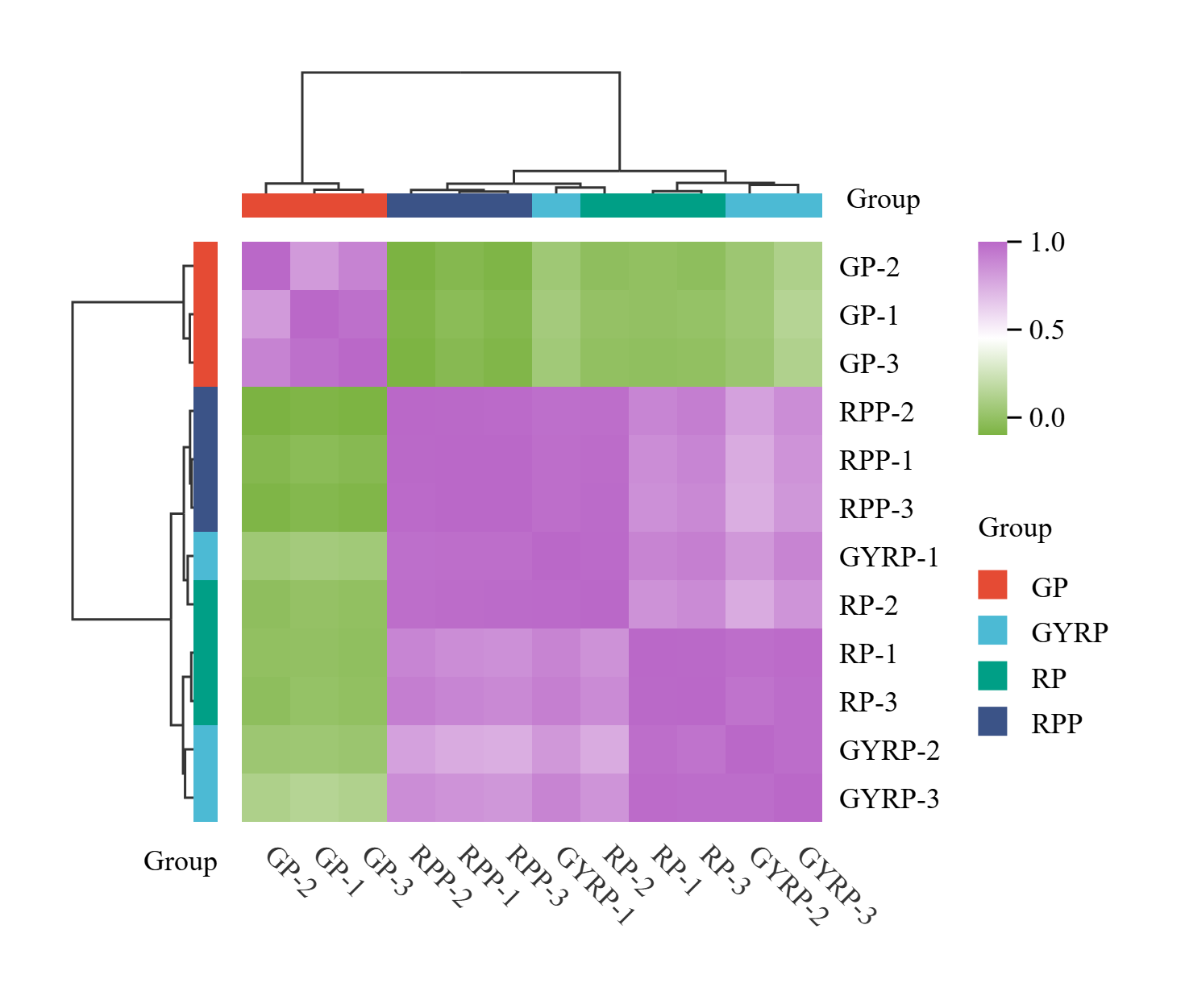

Supplement: Supplementary file 1 [file ijms-25-10754-s001.zip › Figure S1 Pearson Cluster Map.png]

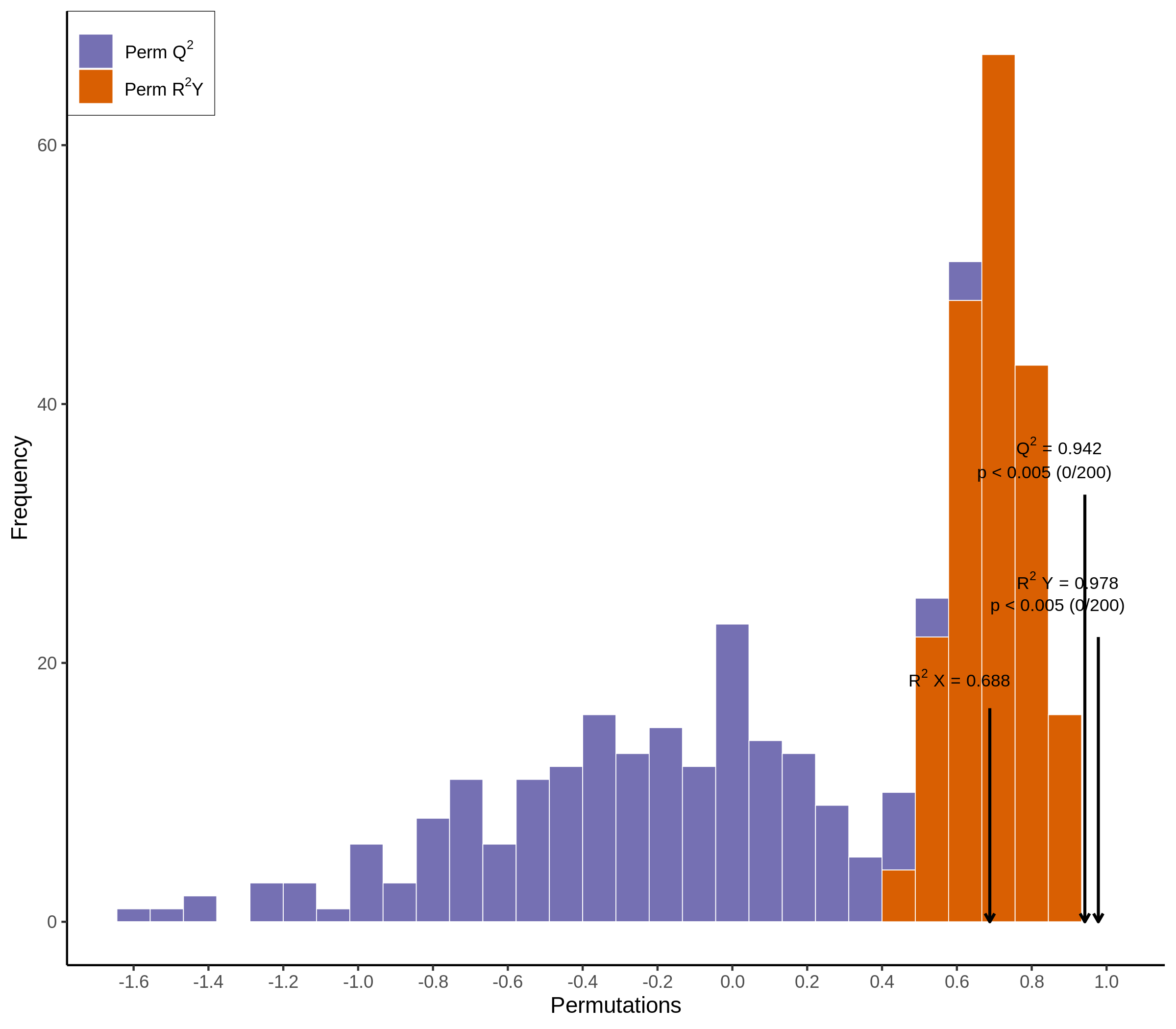

Supplement: Supplementary file 1 [file ijms-25-10754-s001.zip › Figure S2 Result opls permutation.png]

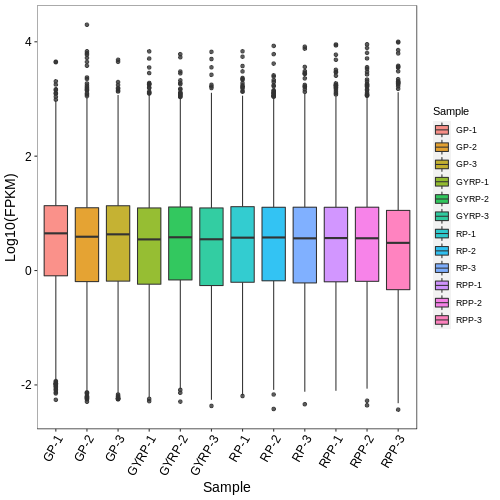

Supplement: Supplementary file 1 [file ijms-25-10754-s001.zip › Figure S3 Box Plot of FPKM Expression Levels Across Samples.png]

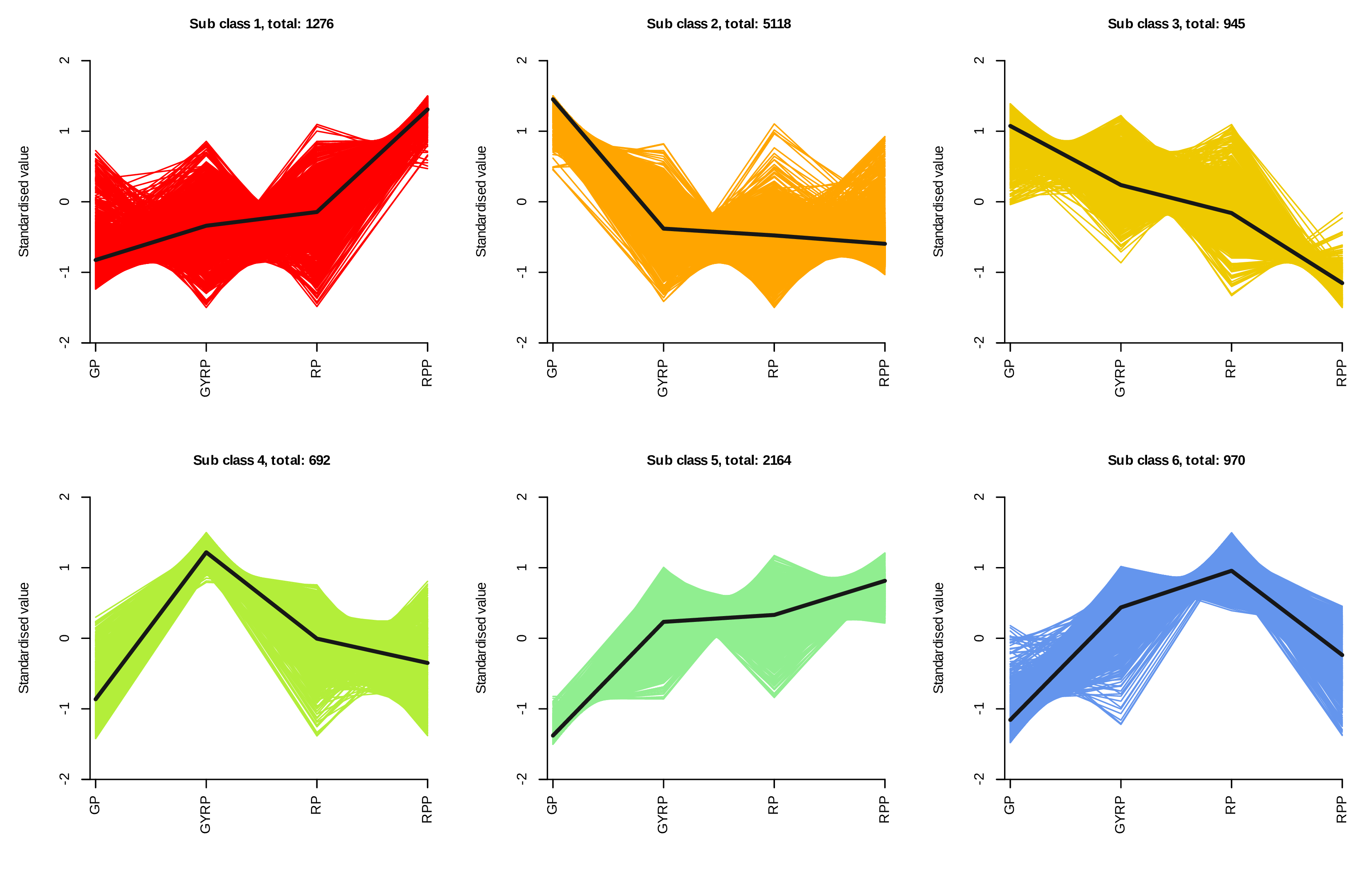

Supplement: Supplementary file 1 [file ijms-25-10754-s001.zip › Figure S4 K-means Clustering Plot.png]

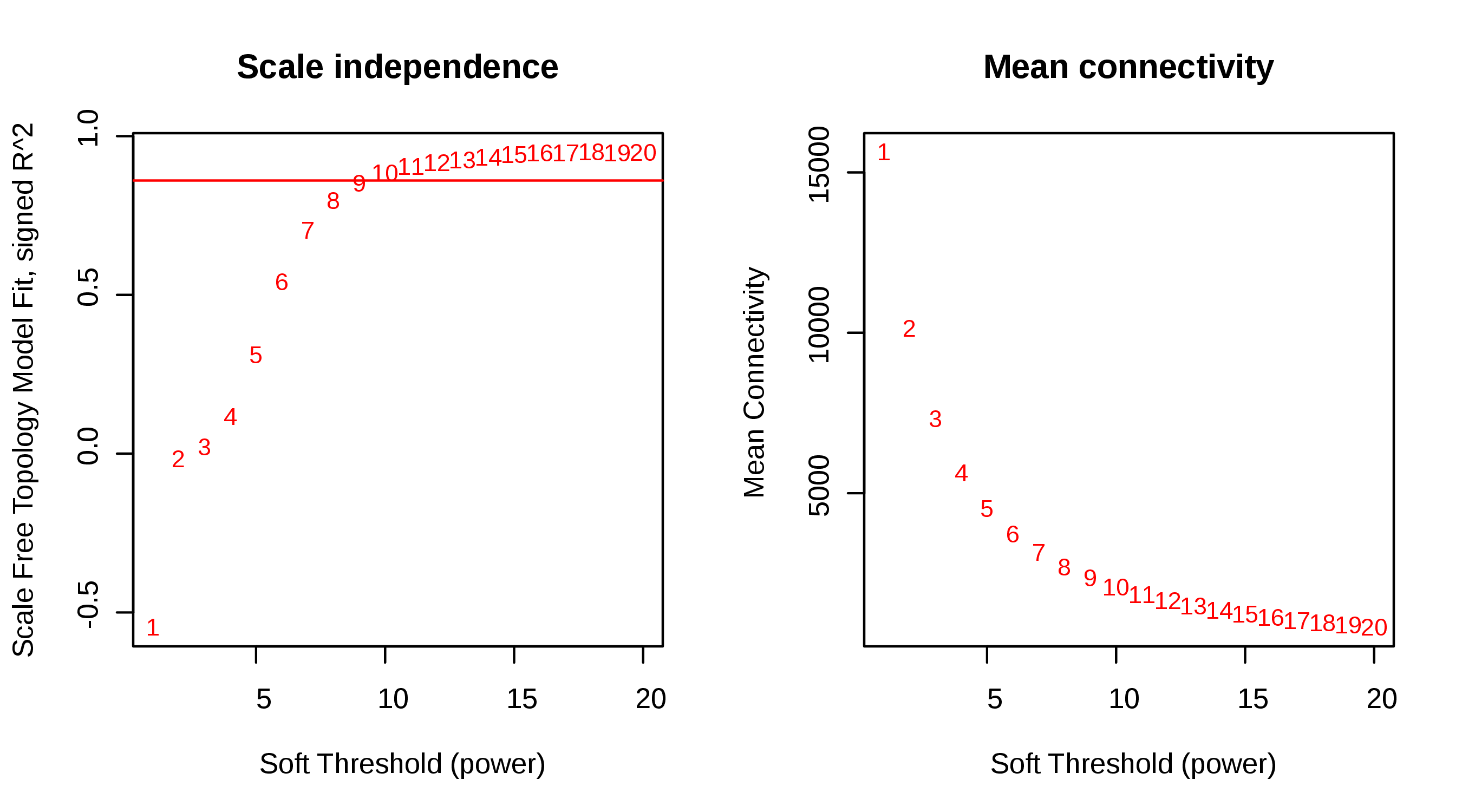

Supplement: Supplementary file 1 [file ijms-25-10754-s001.zip › Figure S5 Diagram of Soft Threshold Selection.png]

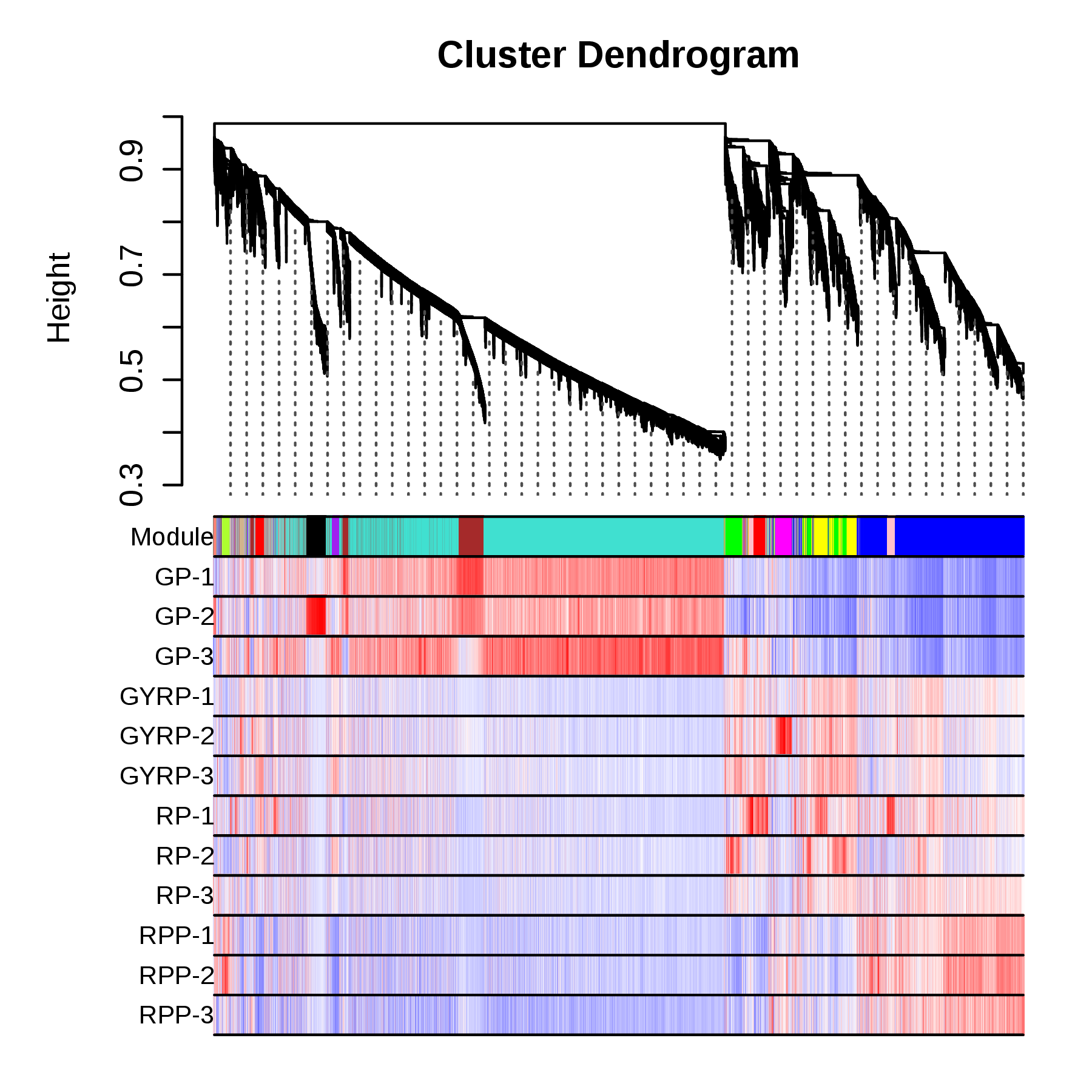

Supplement: Supplementary file 1 [file ijms-25-10754-s001.zip › Figure S6 Heatmap of Gene Clustering Within Modules.png]
